# The French Musculoskeletal Disorders Surveillance Program: Pays de la Loire network

**DOI:** 10.1136/oem.2008.042812

**Published:** 2009-03-05

**Authors:** C Ha, Y Roquelaure, A Leclerc, A Touranchet, M Goldberg, E Imbernon

**Affiliations:** 1 Occupational Health Department, French Institute for Public Health Surveillance, St. Maurice, France; 2 Laboratory of Ergonomics and Occupational Health, IFR 132, Faculty of Medicine, Angers, France; 3 U687, INSERM (National Institute of Health Research), Villejuif, France; 4 Inspection Médicale Régionale du Travail des Pays de la Loire, Nantes, France

## Abstract

**Objectives:**

An epidemiological surveillance system for work-related musculoskeletal disorders (MSDs) was implemented in 2002 in France’s Pays de la Loire region to assess the incidence and prevalence of MSDs in the general and working populations, identify levels of exposure to occupational risk factors and investigate the proportion of cases attributable to work exposure.

**Methods:**

The program combines (1) surveillance of sentinel health events in the general population (carpal tunnel syndrome (CTS) was the sentinel event for upper limb MSDs), (2) assessment of the prevalence of the main upper limb MSDs and their risk factors in the workplace based on a network of occupational physicians and (3) registration of the notification of work-related diseases (WRDs).

**Results:**

1168 incident cases of CTS were included over a 3 year period. The estimated incidence of CTS was 1.00 per 1000 person-years in those aged 20–59 years (0.60 in men and 1.40 in women). The incidence rate was higher in employed than unemployed persons in the year of diagnosis (0.6 per 1000 vs 0.3 in men and 1.7 vs 0.8 in women). The occupational physician network noted high prevalence rates: 11% of men and 15% of women had at least one of the six main upper limb clinically-diagnosed MSDs. The WRD survey showed that MSDs represented 65% of notified WRDs.

**Conclusion:**

The Pays de la Loire program plays a significant role in informing the authorities and the public about the state of current MSDs. It is planned to extend it to a routine national surveillance program.

Musculoskeletal disorders (MSDs) are impairments of bodily structures (such as muscles, tendons, nerves, vessels and cartilage) of the limbs and the back, for example tendinitis and tenosynovitis (mainly shoulder tendinitis, lateral epicondylitis and hand-wrist tendinitis), peripheral nerve entrapment (mainly carpal tunnel syndrome and ulnar tunnel syndrome), bursitis, low back pain and sciatica, and neuro-vascular syndromes (such as vibration white finger). Numerous non-specific peri-articular pain disorders have also been included under this umbrella term.^1^

Work-related MSDs are a leading cause of morbidity and work disability in the European Union^2^ ^3^ and are one of the most worrying issues in occupational health today. They cause considerable human, social and occupational burdens in terms of pain and distress in work and daily life, may lead to irreversible functional after-effects and a reduction in work capacity, and may damage careers. They have steadily increased in the last 15 years and are the main cause of compensated occupational diseases in most industrialised countries. In France, more than 25 000 workers were awarded compensation for limb MSDs by the general National Health Insurance Fund (the organisation that covers workers’ compensation claims for more than 80% of the French population) in 2004, and more than 2700 for chronic lower back disorders, accounting for 81% of all compensated diseases.^4^ These disorders entailed the loss of about 6.3 million days’ work.^4^ MSDs are the main cause of disability before the age of 45 and rank first among the causes of health-related work limitation.^5^ Moreover, they represent an economic challenge because of their effect on manpower, and their constant increase reduces competitiveness. Upper extremity MSDs and low back pain will be increasing problems in the years to come because of the predictable combined effects of ageing of the working population and the intensification of work.

The only source of information available in France to describe the current increased number of MSDs is workers’ compensation claims. However, this is known to be subject to considerable bias and to lead to an underestimation of the extent of the phenomenon.^6^^–^9 As emphasised, accurate surveillance data on MSDs are needed to provide a basis for monitoring changes so as to target industries for additional preventive and regulatory action.^10^ The Occupational Health Department of the French Institute for Public Health Surveillance (DST-InVS) therefore implemented a pilot epidemiological MSD surveillance program in the Pays de la Loire region in 2002.

The aims of this program are: (i) to estimate the incidence and prevalence rates of the main MSDs in the general population of a French region and their time trends according to age, gender, economic sector and occupation; (ii) to assess the levels of the main occupational exposures; (iii) to determine the contribution of work-related physical and psychosocial risk factors to their development; (iv) to estimate the extent of under-declaration of work-related diseases (WRDs) as compensatable occupational diseases; and (v) to assess the feasibility of such a system of surveillance before its extension to other regions in France. The program relies on three main components which were designed to provide a comprehensive overview of the current increased number of MSDs in relation to work. The aim of this paper is to explain the general design of the program and to describe the type of information that each of its components can provide.

## METHODS

This program combines three main components: (1) epidemiological surveillance of sentinel health events in the general population; carpal tunnel syndrome, the most commonly reported nerve entrapment syndrome, was chosen as the sentinel event for upper limb MSDs and sciatica with herniated disk as the sentinel event for back pain; (2) epidemiological surveillance of the main upper limb MSDs and their risk factors in the workplace; and (3) registration of notification data on compensation claims for WRDs related to MSDs. The program was set up in the Pays de la Loire region (Loire valley area, west central France, 3 305 000 inhabitants and 1 247 839 salaried workers in 2002). This region contains 5.5% of the French population and 5.6% of the French working population. Its socioeconomic structure is diversified and close to that of France as a whole.^11^

### Epidemiological surveillance of sentinel health events in the general population

Carpal tunnel syndrome (CTS) results from a compression of the median nerve at the wrist. It represents a major proportion of all registered and/or compensated WRDs in many countries. In France, nearly 40% of workers’ compensation claims for limb MSDs in 2004 were for CTS, a higher proportion than for shoulder and elbow disorders. CTS occupational risk factors are well established and include highly repetitive work, force, the combination of repetitive movements and force, extreme wrist postures and vibration. The purpose of this epidemiological approach was to assess the incidence of CTS according to age, gender, economic sector and occupation. This system of epidemiological surveillance of CTS was tested in the general population of Maine and Loire (one of the five “Departements” of the Pays de la Loire region and containing 1.2% of the French population), aged 20–59 (193 802 men (49.9%) and 194 276 women (50.1%)).^11^ The methods were recently reported in detail.^12^ Incident cases of CTS occurring in residents of Maine and Loire as diagnosed by the four electrodiagnostic (EDX) centres in the region from 2002 to 2004 were included prospectively in the study. All cases were defined by both clinical and electromyographical criteria and the diagnosis was established by neurologists specialised in EDX techniques. The clinical criteria were the classic/probable symptoms according to Katz *et al*^13^: numbness, tingling, burning or pain in at least two of digits 1, 2 or 3; palm pain, wrist pain or pain radiation proximal to the wrist were also included as symptoms for a probable CTS. Case definition agreed with consensus definitions for epidemiological surveillance of CTS and took into account the 2002 AAEM recommendations for the neurophysiological study of CTS.^14^ ^15^ Demographic, clinical and neurophysiological information was provided by the neurologists. A questionnaire was then mailed to each patient. Information was collected on medical and surgical history (obesity, diabetes mellitus, thyroid disease, gynaecological history, upper limb MSDs) and employment during the past 5 years (economic sector and occupation). The final response rate to the questionnaires was 97%.

Incidence rates were estimated per patient (bilateral CTS counting as one case). The date of the electrophysiological examination was used as the date of diagnosis. Age- and gender-specific incidence rates were estimated by dividing the number of subjects with CTS by the number of persons of the same age and gender in the general population according to the 1999 census.^11^ The standardised incidence ratios (SIRs), as estimations of age-adjusted relative risks associated with an economic sector or an occupation, were calculated separately for each gender, using all economic sectors or occupations of the whole sample as reference. The age-adjusted relative risks (RR) of CTS according to economic sectors and occupation categories were computed using the Mantel-Haenszel method with the whole sample of subjects included in the study as reference, whether they were employed during the last 5 years or not. The attributable fractions of CTS among those employed in a certain economic sector or occupation category or subcategory (AFE (%)) were computed to estimate the proportion of CTS cases attributable to work in the economic sectors and occupations at high risk (when at least five cases of CTS occurred) using the following formula: AFE = (RR−1)/RR.^16^

The methods and results from the surveillance of sciatica will be published in the near future.

### Epidemiological surveillance of upper extremity MSDs in the working population

Occupational medicine is a medical speciality in France, and occupational physicians receive 4 years of specialist training. Their tasks include monitoring work exposure and performing annual health examinations, which are compulsory for all salaried workers. Most of the 7000 currently practicing occupational physicians work simultaneously across many companies and industries in the private sector, which employs about 70% of France’s 25 million labour force. The self-employed, civil servants and public sector employees (such as education sector employees) benefit from different occupational medicine arrangements.

To provide data comparable with other European countries, our surveillance protocol followed the recommendations of the Criteria document for evaluating the work-relatedness of upper extremity MSDs, published in 2001 by a group of experts (referred to as the “criteria document” in the remainder of this article).^17^ The aim of the surveillance system is to estimate prevalence rates of MSDs and their risk factors in the regional workforce according to age, gender, economic sector and occupation.

The methods were recently reported in detail.^18^ Briefly, the design was based on a network of occupational physicians. All occupational physicians who practised in this region were invited to participate, and 80 of them (17.4% of the occupational physicians in the region) volunteered to take part in the study. Subjects were randomly selected from workers undergoing a regularly-scheduled annual health examination. The presence of non-specific musculoskeletal symptoms in the upper limbs during the preceding 12 months and the preceding 7 days was identified using the Nordic questionnaire.^19^ If symptoms had occurred during the last 12 months, a physical examination was performed by the physician using a standardised clinical procedure described in the “criteria document” for rotator cuff syndrome, lateral epicondylitis, ulnar tunnel syndrome, CTS, de Quervain’s disease, and flexor–extensor peritendinitis or tenosynovitis of the forearm-wrist region. The final study population comprised 3710 workers (2162 men and 1548 women) employed primarily in manufacturing industries (33%), service industries (25%) or trade (13%), randomly included between April 2002 and April 2005. Women were slightly under-represented in the sample (42% vs 47% in the region). Overall, the distribution of economic sectors and occupations in the study sample was close to that of the regional workforce. Health status was assessed by a self-administered questionnaire and physical examination, and occupational risk factors were assessed by the same questionnaire. Exposure scores were computed for each anatomical area by combining the risk factors described in the “criteria document”.

### Work-related diseases

In France, compensation for an occupational disease is based on a limited number of “tables”. These tables define the medical, technical and administrative conditions that are necessary and sufficient for the financial compensation of an occupational disease. Another category comprises “work-related diseases” (WRDs) defined as diseases considered by the physician to be of occupational origin but not compensatable. The notification of putative WRDs is mandatory for all physicians so that the occupational diseases tables can be updated. However, physicians seldom send in notifications of diseases they believe to be linked to working conditions or occupational exposure.^20^ Moreover, these notifications were not used for epidemiological surveillance due to the lack of information on the source population. The purpose of this part of the program was to estimate the prevalence of WRDs in the working population according to age, gender and economic sector, and to assess the extent of under-declaration of WRDs as a compensatable occupational disease.

A pilot registration system based on 1-week surveys repeated over three periods of 6 months each was implemented in 2003 in the Pays de Loire region with the co-operation of a network of occupational physicians. All occupational physicians who practised in this region were invited to participate. The occupational physicians sent in notifications of the WRDs they observed during the compulsory annual workers’ consultations for each 1-week survey. In order to estimate prevalence rates, we also collected data on age, gender and economic sector for all workers seen by the same occupational physicians during the same period. Nearly half of the region’s occupational physicians volunteered to take part in the pilot stage of the program (about 30% of the region’s occupational physicians participated in each of the three week surveys). They were representative of the region’s occupational physicians in terms of economic sectors covered and were responsible for 339 485 workers in 2003 (about a quarter of all salaried workers in the region).

In all components of the program, economic sector and occupation were coded using the “Nomenclature des Activités Françaises”, the French activities nomenclature (NAF codes), and the “Profession et Catégorie Sociale”, the French classification of occupations (PCS codes).^21^ ^22^

Ethics approval for the program was provided by the French National Commission for Information Processing and Civil Liberties.

## RESULTS

Since the beginning of this program of epidemiological surveillance, several analyses have been performed both for surveillance purposes and to provide information for the public and for health authorities. We here present some results from each component of the program, including some already published, to illustrate its contribution to epidemiological surveillance and research.^23^^–^28

### Incidence of CTS in Maine and Loire

A total of 1168 incident cases (349 men and 819 women) corresponding to 1644 wrists affected by CTS were studied during the 3-year period. A few eligible patients refused to participate but the number of refusals was estimated by the physicians to be less than 10%.

The crude average 12-month incidence of CTS over the 3-year period was 1.00 per 1000 person-years in those aged 20–59 years (0.6 in men and 1.4 in women), increasing with age (p<0.001) in both genders regardless of employment status.

A total of 1135 patients completed the postal questionnaire (320 men, mean age 43.3 years (SD 9.5), and 815 women, mean age 44.9 years (SD 9.4)), most of whom were working at the time of diagnosis (81% of men and 66% of women), and 92.1% (96.9% of men and 90.2% of women) had worked in the last 5 years. The unemployed persons comprised subjects who had never worked (mainly women) and long-term unemployed persons. The crude mean incidence rate of CTS per 1000 person-years was higher in employed persons than in those unemployed in the year of diagnosis (0.6 vs 0.3 in men and 1.7 vs 0.8 in women; both p<0.001).

SIRs varied between economic sectors (table 1). Working in the quarrying industry (mainly sand, stone and clay in the Pays de la Loire region), manufacturing (basic metals, manufacture of metal products, motor vehicles, furniture, wood and wood products, food and beverages industries) and construction was associated with an excess risk in men. Working in agriculture, manufacturing (manufacture of motor vehicles, electrical equipment, chemical industries, food and beverages industries), services (hotels and restaurants, health and social work) and retail trade was associated with an excess of risk in women.

**Table 1:** Standardised incidence ratios of carpal tunnel syndrome according to economic sector (number of observed incident cases ⩾5)

Economic sector (French classification NAF)	Men			Women		
	O	E	SIR (95% CI)	O	E	SIR (95% CI)
A. Agriculture, hunting, forestry and fishing (NAF 01–05)	35	29.8	1.2 (0.8 to 1.6)	80	38.2	2.1 (1.7 to 2.6)
Agriculture (NAF 01)	34	29.2	1.2 (0.8 to 1.6)	80	38.0	2.1 (1.7 to 2.6)
C. Mining and quarrying (stone, sand and clay) (NAF 10–14)	6	1.1	5.3 (1.9 to 11.6)	0	–	–
D. Manufacturing industries (NAF 15–37)	105	69.5	1.5 (1.2 to 1.8)	164	94.9	1.7 (1.5 to 2.0)
Food and drink industry (NAF 15)	22	10.4	2.1 (1.3 to 3.2)	38	14.9	2.5 (1.8 to 3.5)
Garment industry (NAF 18)	1	–	–	13	10.9	1.2 (0.6 to 2.0)
Shoe and leather industry (NAF 19)	11	5.8	1.9 (0.9 to 3.4)	30	22.6	1.3 (0.9 to 1.9)
Manufacture of wood and wood products (NAF 20)	6	2.1	2.9 (1.1 to 6.3)	1	–	–
Manufacture of pulp, paper and paper products (NAF 21)	1	–	–	1	–	–
Publishing, printing and reproduction of recorded media (NAF 22)	2	–	–	5	3.1	1.6 (0.5 to 3.8)
Chemical industry (NAF 24)	2	–	–	12	3.4	3.5 (1.8 to 6.1)
Manufacture of rubber and plastic products (NAF 25)	5	8.8	0.6 (0.2 to 1.3)	6	4.7	1.3 (0.5 to 2.8)
Manufacture of other non-metallic mineral products (NAF 26)	0	–	–	0	–	–
Manufacture of basic metals (NAF 27)	6	1.1	5.6 (2.1 to 12.3)	3	–	–
Manufacture of fabricated metal products (NAF 28)	15	8.5	1.8 (1.0 to 2.9)	7	3.9	1.8 (0.7 to 3.7)
Manufacture of machinery and equipment n.e.c. (NAF 29)	7	7.1	1.0 (0.4 to 2.0)	0	–	–
Manufacture of electrical and optical equipment (NAF 30–33)	8	9.1	0.9 (0.4 to 1.7)	23	14.0	1.6 (1.0 to 2.5)
Manufacture of motor vehicles (NAF 34)	10	2.8	3.6 (1.7 to 6.6)	12	2.8	4.3 (2.2 to 7.5)
Manufacture of other transport equipment (NAF 35)	0	–	–	0	–	–
Manufacture of furniture and wood industries (NAF 36)	9	3.9	2.3 (1.1 to 4.4)	9	4.9	1.8 (0.8 to 3.5)
Recycling (NAF 37)	0	–	–	1	–	–
E. Electricity, gas and water supply (NAF 40–41)	4	–	–	1	–	–
F. Construction (NAF 45)	63	27.7	2.3 (1.8 to 2.9)	6	6.4	0.9 (0.3 to 2.0)
G. Wholesale and retail trade; repair of motor vehicles, motorcycles and personal and household goods (NAF 50–52)	22	33.7	0.7 (0.4 to 1.0)	79	58.2	1.4 (1.1 to 1.7)
Sale, maintenance and repair of motor vehicles and motorcycles (NAF 50)	6	7.2	0.8 (0.3 to 1.8)	8	5.0	1.6 (0.7 to 3.2)
Wholesale trade (NAF 51)	6	14.5	0.4 (0.2 to 0.9)	5	14.0	0.4 (0.1 to 0.8)
Retail trade; repair of personal and household goods (NAF 52)	10	12.0	0.8 (0.4 to 1.5)	66	39.3	1.7 (1.3 to 2.1)
H. Hotels and restaurants (NAF 55)	4	–	–	26	15.8	1.6 (1.1 to 2.4)
I. Transport, storage and communication (NAF 60–64)	16	16.0	1.0 (0.6 to 1.6)	13	13.0	1.0 (0.5 to 1.7)
Land transport (NAF 60)	6	8.5	0.7 (0.3 to 1.5)	2	–	–
Auxiliary transport activities (NAF 63)	2	–	–	3	–	–
Post and telecommunications (NAF 64)	8	5.3	1.5 (0.7 to 3.0)	8	8.6	0.9 (0.4 to 1.8)
J. Financial intermediation, insurance and pension funding (NAF 65–67)	0	–	–	15	16.6	0.9 (0.5 to 1.5)
Insurance and pension funding, except compulsory social security (NAF 66)	0	–	–	7	4.1	1.7 (0.7 to 3.5)
K. Real estate, renting and business activities (NAF 70–74)	6	22.7	0.3 (0.1 to 0.6)	33	36.3	0.9 (0.6 to 1.3)
Other business activities (legal. accounting, advertising…) (NAF 74)	5	16.3	0.3 (0.1 to 0.7)	25	27.3	0.9 (0.6 to 1.4)
L. Public administration, compulsory social security (NAF 75)	17	19.7	0.9 (0.5 to 1.4)	39	51.8	0.8 (0.5 to 1.0)
M. Education (NAF 80)	10	15.0	0.7 (0.3 to 1.2)	62	65.9	0.9 (0.7 to 1.2)
N. Health and social work (NAF 85)	10	12.7	0.8 (0.4 to 1.5)	164	115.8	1.4 (1.2 to 1.7)
O. Social and personal service activities (NAF 90–93)	7	7.8	0.9 (0.4 to 1.8)	25	23.5	1.1 (0.7 to 1.6)
P. Household activities (NAF 95)	0	–	–	19	16.4	1.2 (0.7 to 1.8)

E, expected; NAF, “Nomenclature des Activités Françaises”, the French activities nomenclature code; n.e.c., not elsewhere classified; O, observed; SIR, standardised incidence ratio.

In terms of occupation categories (table 2), the workers affected by CTS belonged mainly to lower grade white collar and blue collar categories including material handlers, unskilled industrial, craft and agricultural workers of both genders and skilled male craft workers. The categories for female workers were mainly trade and commerce employees (cashiers, food sales employees, self-service store employees), personal services employees (restaurant or café waitresses, hairdressers, domestic cleaners, child care workers), government executives and service workers (nursing auxiliaries, school cleaners, hospital cleaners).

**Table 2:** Standardised incidence ratios of carpal tunnel syndrome according to occupation (number of observed incident cases ⩾5)

Occupation (French classification PCS)		Observed	Expected	SIR (95% CI)
Women				
1. Farmers (PCS 11–13)		19	21.8	0.9 (0.5 to 1.4)
2. Craftsmen, salesmen, employers (PCS 21–23)		10	24.2	0.4 (0.2 to 0.8)
3. Managers and professionals (PCS 31–38)		23	38.5	0.6 (0.4 to 0.9)
4. Teaching associate professionals, health and social work intermediate occupations,		64	115.0	0.6 (0.4 to 0.7)
administrative intermediate occupations of public and private companies, technicians				
and associate professionals, supervisors (PCS 42–48)				
5. Employees and clerks (PCS 52–56)		384	252.1	1.5 (1.4 to 1.7)
Government executive officials and service workers (PCS 52)		141	90.3	1.6 (1.3 to 1.8)
Government executive officials (PCS 5215)		10	3.4	2.9 (1.4 to 5.4)
Nursing auxiliaries (PCS 5221)		38	18.2	2.1 (1.5 to 2.9)
School cleaners and related cleaners (PCS 5216)		26	13.3	2.0 (1.3 to 2.9)
Hospital cleaners (PCS 5222)		32	18.2	1.8 (1.2 to 2.5)
Trade and commerce employees (PCS 55)		61	23.0	2.7 (2.0 to 3.4)
Shop cashiers (PCS 5519)		18	4.4	4.1 (2.4 to 6.5)
Food sales employees (PCS 5512)		12	5.0	2.4 (1.2 to 4.2)
Self-service store employees (PCS 5518)		11	4.6	2.4 (1.2 to 4.2)
Personal services employees (PCS 56)		117	69.1	1.7 (1.4 to 2.0)
Restaurant or café waitresses (PCS 5611)		16	5.2	3.1 (1.7 to 5.0)
Hairdressers (PCS 5622)		6	2.0	3.0 (1.1 to 6.5)
Child care workers (PCS 5631)		64	41.9	1.5 (1.2 to 2.0)
6. Skilled and unskilled workers (PCS 62–69)		234	102.6	2.3 (2.0 to 2.6)
Material handlers and related equipment workers (PCS 65)		11	1.8	6.2 (3.1 to 11.1)
Storekeepers (PCS 6515)		8	1.5	5.2 (2.2 to 10.2)
Unskilled industrial workers (PCS 67)		129	43.3	3.0 (2.5 to 3.5)
Agricultural and food industries (PCS 6754)		24	3.6	6.7 (4.3 to 10.0)
Sorting. packaging and dispatch (PCS 6793)		23	3.3	7.0 (4.4 to 10.5)
Mechanical machinery assemblers (PCS 6723)		13	2.1	6.1 (3.3 to 10.5)
Electrical and electronic equipment assemblers (PCS 6711)		14	4.6	3.1 (1.7 to 5.2)
Clothing industry (PCS 6772)		12	5.2	2.3 (1.2 to 4.0)
Shoe and leather work (PCS 6773)		19	11.0	1.7 (1.0 to 2.7)
Unskilled craft workers (PCS 68)		23	12.6	1.8 (1.2 to 2.7)
Cleaners (PCS 6891)		19	9.3	2.0 (1.2 to 3.2)
Unskilled agricultural workers (PCS 69)		48	12.4	3.9 (2.9 to 5.1)
Orchard and vineyard (PCS 6914)		17	2.5	6.9 (4.0 to 11.1)
Breeding workers (PCS 6912)		7	1.3	5.2 (2.1 to 10.8)
Growing of vegetables, horticultural specialities (PCS 6913)		24	6.6	3.6 (2.3 to 5.4)
Men				
1. Farmers (PCS 11–13)		14	18.5	0.8 (0.4 to 1.3)
2. Craftsmen, salesmen, employers (PCS 21–23)		13	25.3	0.5 (0.3 to 0.9)
3. Managers and professionals (PCS 31–38)		17	34.8	0.5 (0.3 to 0.8)
4. Teaching associate professionals, health and social work intermediate occupations,		29	57.2	0.5 (0.3 to 0.7)
administrative intermediate occupations of public and private companies, technicians				
and associate professionals, supervisors (PCS 42–48)				
5. Employees and clerks (PCS 52–56)		24	24.4	1.0 (0.6 to 1.5)
6. Skilled and unskilled workers (PCS 62–69)		210	110.0	1.9 (1.7 to 2.2)
Skilled craft workers (PCS 63)		55	25.9	2.1 (1.6 to 2.8)
Plumbers (PCS 6344)		5	1.5	3.3 (1.1 to 7.7)
Gardeners (PCS 6301)		6	1.8	3.3 (1.2 to 7.1)
Bricklayers (PCS 6341)		9	4.6	1.9 (0.9 to 3.7)
Material handlers and related equipment workers (PCS 65)		21	6.1	3.4 (2.1 to 5.2)
Lift truck drivers (PCS 6514)		7	2.2	3.1 (1.3 to 6.5)
Storekeepers (PCS 6515)		10	3.4	2.9 (1.4 to 5.4)
Unskilled industrial workers (PCS 67)		59	21.1	2.8 (2.1 to 3.6)
Mechanical machinery assemblers (PCS 6723))		15	2.2	6.7 (3.7 to 11.0)
Agricultural and food industries (PCS 6754)		11	2.0	5.5 (2.7 to 9.8)
Shoe and leather work (PCS 6773)		6	2.0	3.0 (1.1 to 6.6)
Unskilled craft workers (PCS 68)		22	8.5	2.6 (1.6 to 3.9)
Building construction (PCS 6841)		12	1.7	7.1 (3.7 to 12.4)
Building installation and completion (PCS 6842)		6	1.7	3.5 (1.3 to 7.6)
Unskilled agricultural workers (PCS 69)		20	8.6	2.3 (1.4 to 3.6)
Breeding workers (PCS 6912)		4	–	–
Orchard and vineyard (PCS 6914)		10	2.7	3.7 (1.8 to 6.8)

PCS, “Profession et Catégorie Sociale”, the French classification of occupations; SIR, standardised incidence ratio.

The attributable fractions of CTS to work among exposed persons (AFE) in occupations at high risk showed that a substantial proportion of cases of CTS diagnosed in lower grade white collar and blue collar workers were attributable to work: AFEs reached higher values in female blue collar workers (67%) and lower-grade services, sales and clerical white collar workers (61%). The AFE in male blue collar workers was 76%. High AFE values were observed in agriculture (58% for women), manufacturing industries (from 58% to 92% for men and from 42% to 93% for women), construction (66% for men), personal service activities (66% for women), trade and commerce (49% for women) and hotels and restaurants (44% for women).^28^

### Epidemiological surveillance of upper extremity MSDs in the working population

Among the 3710 workers randomly included between April 2002 and April 2005, 52% of men and 57% of women had experienced upper extremity non-specific musculoskeletal symptoms during the preceding 12 months, and 27% of men and 35% of women had experienced these symptoms during the preceding week. Prevalence rates of clinically-diagnosed MSDs were high for both genders: 645 cases were diagnosed in 521 workers. A total of 11.2% (95% CI 9.9% to 12.6%) of men and 14.8% (95% CI 13.0% to 16.6%) of women had at least one of the six main upper extremity MSDs. The most frequent disorder was rotator cuff syndrome (6.6% (95% CI 5.6% to 7.6%) in men and 8.5% (95% CI 7.1% to 9.9%) in women), followed by CTS (2.4% (95% CI 1.8% to 3.0%) in men and 4.0% (95% CI 3.0% to 5.0%) in women) and lateral epicondylitis (2.4% (95% CI 1.8% to 3.0%) in men and 2.5% (95% CI 1.7% to 3.3%) in women). Prevalence rates of the three other MSDs were lower at <1%, except for De Quervain’s disease in women at 2.1% (95% CI 1.4% to 2.8%). Prevalence rates increased with age for both genders, even after adjustment on seniority (p<0.001). After the age of 50, 18% of men and 22% of women had at least one MSD and 3.2% of men and 5.6% of women had at least two MSDs.

Prevalence rates of MSDs varied between economic sectors (table 3). In men, prevalence rates were highest in the automotive industries (NAF code: 34), manufactured metal products (28), public administration (75), electrical and optical equipment (30–33), machine and equipment industries (29) and rubber and plastic product industries (25). In women, the highest prevalence rates were found in agriculture, several manufacturing industries (rubber and plastic industries (25), paper industry (21), garment industry (18), machine and equipment industry (29), furniture and wood industries (36)), and also in public administration (75), post and telecommunications (64) and hotels and restaurants (55).

**Table 3:** Sex-specific prevalence rates of at least one clinically diagnosed upper limb musculoskeletal disorder according to economic sector of employment

Economic sector (French classification NAF)	Men			Women		
	No	n	%	No	n	%
A. Agriculture, hunting, forestry and fishing (NAF 01–05)	31	2	6.5	25	7	28.0
Agriculture (NAF 01)	31	2	6.5	25	7	28.0
C. Mining and quarrying (stone, sand and clay) (NAF 10–14)	18	3	16.7	6	2	33.3
D. Manufacturing industries (NAF 15–37)	829	107	12.9	395	77	19.5
Food and drink industry (NAF 15)	182	22	12.1	113	15	13.3
Garment industry (NAF 18)	1	0	0.0	12	4	33.3
Shoe and leather industry (NAF 19)	8	0	0.0	28	3	10.7
Manufacture of wood and wood products (NAF 20)	24	3	12.5	6	3	50.0
Manufacture of pulp, paper and paper products (NAF 21)	52	6	11.5	12	4	33.3
Publishing, printing and reproduction of recorded media (NAF 22)	17	1	5.9	9	1	11.1
Chemical industry (NAF 24)	8	0	0.0	2	1	50.0
Manufacture of rubber and plastic products (NAF 25)	84	12	14.3	45	15	33.3
Manufacture of other non-metallic mineral products (NAF 26)	22	1	4.6	2	0	0.0
Manufacture of basic metals (NAF 27)	23	2	8.7	6	3	50.0
Manufacture of fabricated metal products (NAF 28)	91	17	18.7	11	2	18.2
Manufacture of machinery and equipment n.e.c. (NAF 29)	89	13	14.6	26	6	23.1
Manufacture of electrical and optical equipment (NAF 30–33)	89	7	15.7	69	10	14.5
Manufacture of motor vehicles (NAF 34)	63	16	25.4	2	0	0.0
Manufacture of other transport equipment (NAF 35)	9	1	11.1	2	1	50.0
Manufacture of furniture and wood industries (NAF 36)	57	6	10.5	45	8	17.8
Recycling (NAF 37)	7	0	0.0	–	–	–
E. Electricity, gas and water supply (NAF 40–41)	12	2	16.7	3	1	33.3
F. Construction (NAF 45)	189	25	13.2	25	1	4.0
G. Wholesale and retail trade; repair of motor vehicles, motorcycles and personal and household goods (NAF 50–52)	240	20	8.3	238	31	13.0
Sale, maintenance and repair of motor vehicles and motorcycles (NAF 50)	62	5	8.1	11	2	18.2
Wholesale trade (NAF 51)	96	9	9.4	50	7	14.0
Retail trade, repair of personal and household goods (NAF 52)	82	6	7.3	177	22	12.4
H. Hotels and restaurants (NAF 55)	40	1	2.5	47	7	14.9
I. Transport, storage and communication (NAF 60–64)	152	20	13.2	67	10	14.9
Land transport (NAF 60)	63	8	12.7	19	3	15.8
Auxiliary transport activities (NAF 63)	13	2	15.4	5	0	0.0
Post and telecommunications (NAF 64)	75	10	13.3	42	7	16.7
J. Financial intermediation, insurance and pension funding (NAF 65–67)	74	9	12.2	76	7	9.2
Insurance and pension funding, except compulsory social security (NAF 66)	54	7	13.0	54	6	11.1
K. Real estate, renting and business activities (NAF 70–74)	267	17	6.4	196	20	10.2
Other business activities (legal, accounting, advertising, etc) (NAF 74)	229	15	6.6	163	18	11.0
L. Public administration, compulsory social security (NAF 75)	174	29	16.7	140	25	17.9
M. Education (NAF 80)	15	0	0.0	20	3	15.0
N. Health and social work (NAF 85)	72	4	5.6	236	30	12.7
O. Social and personal services activities (NAF 90–93)	44	4	9.1	72	8	11.1
P. Activities of households (NAF 95)	–	–	–	1	0	0.0
Total	2157	243	11.3	1544	228	14.8

No, number of subjects in the study; n, number of cases; NAF, “Nomenclature des Activités Françaises”, the French activities nomenclature code; n.e.c., not elsewhere classified.

Prevalence rates of MSDs varied according to occupation (table 4). For men, the highest prevalence rates were observed for public service employees (for example, policemen and members of the armed forces) (PCS code: 51–53), skilled and unskilled industrial workers (62, 67) and storekeepers (65). For women, the highest prevalence rates were observed for agricultural workers (69), skilled craft workers (63), skilled and unskilled industrial workers (62, 67), personal services employees (56) (personal care employees), and public service employees (51–53).

**Table 4:** Specific prevalence rates of at least one clinically diagnosed upper limb MSD according to occupation

Occupation (French classification PCS)	Men			Women		
	No	n	%	No	n	%
3. Managers and professionals (PCS 31–38)	210	18	8.6	78	6	7.7
Administrative managers (PCS 32–35)	42	3	7.1	25	3	12.0
Directors and chief executives (PCS 37)	89	6	6.7	33	3	9.1
Production and operations department managers (PCS 38)	78	9	11.5	16	0	0
4. Associate professionals and technicians (PCS 41–48)	540	49	9.1	289	39	13.5
Teaching, public services and health associate professionals (PCS 41–43, 45)	96	8	8.3	137	20	14.6
Administrative and commercial associate professionals (PCS 46)	110	7	6.4	101	12	11.9
Technicians (PCS 47)	209	21	10.0	32	4	12.5
Supervisors (PCS 48)	123	13	10.6	19	3	15.8
5. Employees and clerks (PCS 51–56)	188	16	8.5	798	98	12.3
Public services employees (PCS 51–53)	80	13	16.3	219	35	16.0
Administrative employees and clerks (PCS 54)	48	0	0	328	28	8.5
Trade and commerce employees (PCS 55)	38	3	7.9	147	17	11.6
Personal services employees (PCS 56)	22	0	0	104	18	17.3
6. Skilled and unskilled workers (PCS 61–69)	1209	158	13.1	377	84	22.3
Skilled workers (PCS 61–65)	832	111	13.3	111	24	21.6
Skilled industrial workers (PCS 62)	347	47	13.5	61	12	19.7
Skilled craft workers (PCS 63)	254	30	11.8	17	7	41.2
Drivers (PCS 64)	102	12	11.8	17	3	17.6
Storekeepers (PCS 65)	129	22	17.1	16	2	12.5
Unskilled workers (PCS 67–69)	377	47	12.5	266	60	22.6
Unskilled industrial workers (PCS 67)	273	39	14.3	206	45	21.8
Unskilled craft workers (PCS 68)	71	6	8.5	39	5	12.8
Agricultural workers (PCS 69)	33	2	6.1	21	10	47.6
Total	2160	243	11.3	1545	229	14.8

No, number of subjects in the study; n, number of cases; PCS, “Profession et Catégorie Sociale”, the French classification of occupations.

High numbers of workers were exposed to at least two risk factors for MSDs of the neck (43%), shoulder (44%), elbow (50%) and wrist (60%). Excluding the neck, only 10% of workers were free of exposure to any of the 17 biomechanical or psychosocial risk factors listed in the “criteria document”; 25% were exposed to one risk factor, 66% to two or more risk factors, 39% to at least four and 9% to seven or more. According to the “criteria document”, a high percentage of cases of MSDs could be classified as “probably work-related” (85% in women and 89% in women <50 years of age, and 87% in men and 90% in men <50 years of age). Exposure varied according to economic sector and occupation. Exposure was particularly high in quarrying industries, several manufacturing industries, agriculture and construction. The most exposed workers were unskilled craft and industrial workers, skilled craft and industrial workers, storekeepers and agricultural workers.

### Work-related diseases

For 23 416 workers seen in compulsory medical consultations during the 3 weeks of the pilot study (October 2003, April and October 2004), 1056 notifications of WRDs were recorded. Work-related MSDs (upper and lower limb disorders, back pain) accounted for 65.1% of these WRDs, followed by mental and behavioural or psychological disorders (24.0%), skin diseases (4.9%), hearing problems (2.5%) and diseases of the respiratory system (1.9%). The prevalence of WRDs was 4.6% of total diseases or symptoms, and 2.9% of work-related MSDs.

According to the occupational physicians, 61% of the work-related MSDs could have been claimed as a compensatable occupational disease according to the conditions defined in the tables. Only 11% (46 cases) of these 417 workers claimed compensation for an occupational disease. The absence of MSD claims for a compensatable occupational disease was explained in 43% of cases by the employee’s refusal to claim, and in the remainder by recently diagnosed disease and lack of information about rights on the part of the employee or the physician. The body regions mostly affected in men were the lower back (32%), shoulder (25%), elbow and hand-wrist (19%), and in women the hand-wrist (34%), shoulder (32%), lower back (20%) and elbow (17%).

The economic sectors with the highest prevalence rates of MSDs were identified for each gender separately. The highest prevalence rates for men occurred in mining and quarrying, agriculture, construction and several manufacturing industries (pulp and paper, food and drink, furniture, non-metallic mineral products, motor vehicles, rubber and plastic products, machines and electrical equipment industries) and also in professional, business, religious and political organisations, etc.

The highest prevalence rates for women were observed in agriculture and in some of the same manufacturing industries as men (rubber and plastics, machines and electrical equipment, pulp and paper, food and drink, furniture and automotive industries) and in others such as manufacturing of wood and wood products, clothing products, shoes and leather products.

## DISCUSSION

The program described here is an epidemiological surveillance system intended to provide information on the trends and course of the current increased number of MSDs on a population scale.

The CTS surveillance results provided the first estimation of the frequency of this MSD in the general population of a French district. This district is characterised by highly developed manufacturing and meat and poultry industries and by certain types of specialised cultivation (vineyards, horticulture and arboriculture). The main limitation of the study was the lack of exhaustiveness of the surveillance network of neurologists, which led to an underestimation of the incidence of CTS.^12^ ^28^

The male/female ratio in the rate of CTS per 1000 person-years was similar between employed (0.35) and unemployed persons (0.37). This result could indicate that non-occupational factors favouring a higher incidence in women have a similar influence in both genders. AFE values were of the same order of magnitude for both genders in blue collar workers and manufacturing industries, suggesting that occupational factors have the same influence in men and women. The results showed variations according to sectors and occupations, in agreement with the results of Rossignol *et al* in Quebec.^29^ Our study identified 14 sectors with an excess risk of CTS: agriculture, quarrying, construction, manufacturing (food and drink industry, wood and wood products, chemical industries, basic metals and metal products, motor vehicles, electrical or optical equipment and electronic components, furniture), retail trade, hotels and restaurants, health and social work. These sectors account for approximately 22% of male employment and approximately 44% of female employment in Maine and Loire. Since many tests have been performed, we are aware that some of the significant results might be due to random effects.

**Main messages**

- The French Musculoskeletal Disorders (MSDs) Surveillance Program was implemented in 2002 by the French Institute for Public Health Surveillance.
- This program, first implemented in the Pays de la Loire region, is being extended to other regions of France.
- The crude mean incidence rate of carpal tunnel syndrome (CTS) per 1000 person-years was higher in employed than in unemployed persons in the year of diagnosis (0.6 vs 0.3 in men and 1.7 vs 0.8 in women). The attributable fractions of CTS to work among exposed persons were higher in blue collar and lower-grade white collar workers.
- Prevalence rates of clinically-diagnosed upper limb MSDs were high for both genders (11.2% of male workers and 14.8% of female workers).
- According to the occupational physicians, workers claimed for only a small number of the 61% of work-related MSDs that were compensatable occupational diseases.

**Policy implication**

The French Musculoskeletal Disorders (MSDs) Surveillance Program in Pays de la Loire has already played a significant role in informing the authorities and the public on the state of the current increased number of MSDs in France and should be extended to other regions.

Surveillance of the main MSDs in the working population revealed high prevalence rates for clinically diagnosed MSDs: 11% (95% CI 10% to 13%) of men and 15% (95% CI 13% to 17%) of women had one of the six upper limb MSDs and about 2% had at least two disorders. Less than 5% of eligible workers refused to participate. The high prevalence of clinically diagnosed MSDs contrasted with the relatively low level of workers’ compensation claims for upper limbs MSDs in the same region (about 3.7 workers’ compensation claims per 1000 workers in 2003). This confirms that in France, as in other countries, using workers’ compensation claims as a source of information leads to underestimation of the frequency of MSDs. Contrary to French and regional workers’ compensation claims figures, the most prevalent disorder was not CTS but rotator cuff syndrome. The high prevalence rate of this disorder is worrying because of its poor medical and social prognosis.

Another finding that is of importance for the prevention of MSDs was the high prevalence rate after 50 years of age. The accumulation of MSDs in older employees probably reduces their functional capacity and increases the risk of disability and early retirement.

The data generated by the epidemiological surveillance program were also used for research work. The feasibility of a job exposure matrix for exposure assessment in studies of work-related MSDs of the upper extremities was explored.^24^ The factors associated with excess risk of upper limb MSDs in manual workers compared with other workers were investigated, and the variables which best summarised biomechanical exposure associated with upper extremity disorders were identified.^25^ ^27^

Improved registration of WRDs was observed during the 3 weeks of the pilot study, yielding more than 1000 notifications instead of 845 and 536 for the whole of 2001 and 2002, respectively, for all occupational physicians in the region. If repeated regularly, these short periods of registration should provide valuable information on the frequency of work-related pathological disorders, as well as an estimate of the extent of undeclared compensatable occupational diseases.

### Geographical extension

The implementation of this pilot program of epidemiological surveillance of MSDs over 3 years (2002–2004) in the Pays de la Loire region identified the strengths and limitations of several sources of data and of different methods of collection. Based on that experience, we are currently preparing to extend it geographically within the framework of a national program whose main objectives remain those of the Pays de la Loire pilot study. The program is being extended through its progressive implementation in several other regions chosen as representative of French economic activity. The surveillance methods have been simplified for this extension. For the surveillance of sentinel health events in the general population, the program will rely on the use of data from the French national hospital database for surgical cases. For surveillance of the working population, a simplified self-administered questionnaire will be completed by a sample of workers, and only the three most frequent MSDs of the upper limb (shoulder tendinitis, CTS and lateral epicondylitis) will be investigated through a standardised clinical examination by occupational physicians. For WRD surveillance, week-long surveys will be performed regularly.

The first region to which the program is currently being extended is Provence-Alpes-Côte d’Azur (southeastern France), which was chosen because the structure of the sectors of economic activity is quite different from that of the Pays de la Loire region (fewer manufacturing industries, more services sectors). The surveillance of WRDs has already been implemented in seven other regions, covering about 29% of the French population. Extension of the data collection to the rest of the country will clarify and strengthen the results observed in only one region and increase the probability of decision-makers looking seriously at the problems with this system. Different selection biases may be introduced by variations in the participating rates of occupational physicians according to region. These potential selection biases will be explored when data are available.

The Pays de la Loire experimental program has already played a significant role in informing the authorities and the public of the state and course of the current increased number of MSDs, although the data gathered have still to be fully exploited. We plan to repeat our surveillance periodically; the next step will be conducted in 2011–2012 to study time trends. To our knowledge, such a program is unique, and we intend to develop it into a routine national epidemiological MSD surveillance program.
